# A molecule-like PtAu_24_(SC_6_H_13_)_18_ nanocluster as an electrocatalyst for hydrogen production

**DOI:** 10.1038/ncomms14723

**Published:** 2017-03-10

**Authors:** Kyuju Kwak, Woojun Choi, Qing Tang, Minseok Kim, Yongjin Lee, De-en Jiang, Dongil Lee

**Affiliations:** 1 Department of Chemistry, Yonsei University, Seoul 03722, South Korea; 2 Department of Chemistry, University of California, Riverside, California 92521, USA

## Abstract

The theoretically predicted volcano plot for hydrogen production shows the best catalyst as the one that ensures that the hydrogen binding step is thermodynamically neutral. However, the experimental realization of this concept has suffered from the inherent surface heterogeneity of solid catalysts. It is even more challenging for molecular catalysts because of their complex chemical environment. Here, we report that the thermoneutral catalyst can be prepared by simple doping of a platinum atom into a molecule-like gold nanocluster. The catalytic activity of the resulting bimetallic nanocluster, PtAu_24_(SC_6_H_13_)_18_, for the hydrogen production is found to be significantly higher than reported catalysts. It is even better than the benchmarking platinum catalyst. The molecule-like bimetallic nanocluster represents a class of catalysts that bridge homogeneous and heterogeneous catalysis and may provide a platform for the discovery of finely optimized catalysts.

## Abstract (web summary)

Volcano plots for electrocatalytic hydrogen production show the best catalysts as those ensuring the hydrogen binding step is thermodynamically neutral. Here, the authors report fabrication of a highly active thermoneutral electrocatalyst via doping of a single platinum atom into a gold nanocluster.

Hydrogen (H_2_) has been considered as an alternative energy carrier and generated the intense interest in creating artificial catalytic systems that can efficiently produce H_2_ from water^1^,^2^,^3^,^4^. Hydrogenase enzymes that contain iron and nickel cofactors are efficient hydrogen evolution reaction (HER) catalysts with turnover frequencies (TOFs) as high as 9,000 mol of H_2_ per mole of catalyst per second (mol H_2_ (mol cat)^−1^ s^−1^)^5^. There have been a variety of synthetic molecular catalysts, including nickel, cobalt, iron and molybdenum complexes, to mimic the biological catalysts^6^,^7^,^8^,^9^,^10^,^11^,^12^. However, the TOF values reported for these complexes remain quite low (<4.1 mol H_2_ (mol cat)^−1^ s^−1^), and they often show limited stability in aqueous media^13^. Moreover, practical electrocatalysis typically requires their immobilization on electrode materials that can operate fully in aqueous media^14^,^15^. Recent progress in the computational design of solid catalysts has revealed the importance of engineering of the structure and adsorption energies for catalysis at surfaces^16^,^17^,^18^. In fact, some engineered surface alloys exhibit high catalytic activities comparable to platinum^18^. However, the inherent heterogeneity in the surface structure and composition often found in these solid catalysts hamper the fine controlling of these properties at the molecular scale.

Ligand-protected gold and silver nanoclusters have received much attention as materials with many practical applications because of promises offered by their unique optical, electrochemical and catalytic properties^19^,^20^,^21^,^22^. Unlike the supported cluster systems^23^,^24^, these clusters are stable without the support and readily soluble in appropriate solvents. They have special stability at certain compositions, and thus molecule-like clusters with precise compositions are typically obtained from various size-controlled syntheses^21^,^22^. Au_25_(SR)_18_, Au_102_(SR)_44_ and Ag_44_(SR)_30_, where SR is a thiolate ligand, are the examples of these clusters whose stability is thought to arise from the closure of superatomic electron shells^25^,^26^,^27^. While stable clusters to date are rather limited to Au and Ag systems, bimetallic clusters prepared by doping a foreign metal into the stable Au and Ag clusters have opened an avenue to the fine-tuning of the cluster properties^28^,^29^,^30^,^31^,^32^,^33^. Here we report a highly active HER catalyst based on a molecule-like bimetallic cluster that can generate H_2_ with TOFs of 4.8 in tetrahydrofuran (THF) and 34 mol H_2_ (mol cat)^−1^ s^−1^ in water, respectively, at a moderate overpotential (*η*=0.6 V). This cluster catalyst represents a special type of catalyst that bridges homogeneous and heterogeneous catalysis.

## Results

### Molecule-like metal nanoclusters

**Figure 1:**
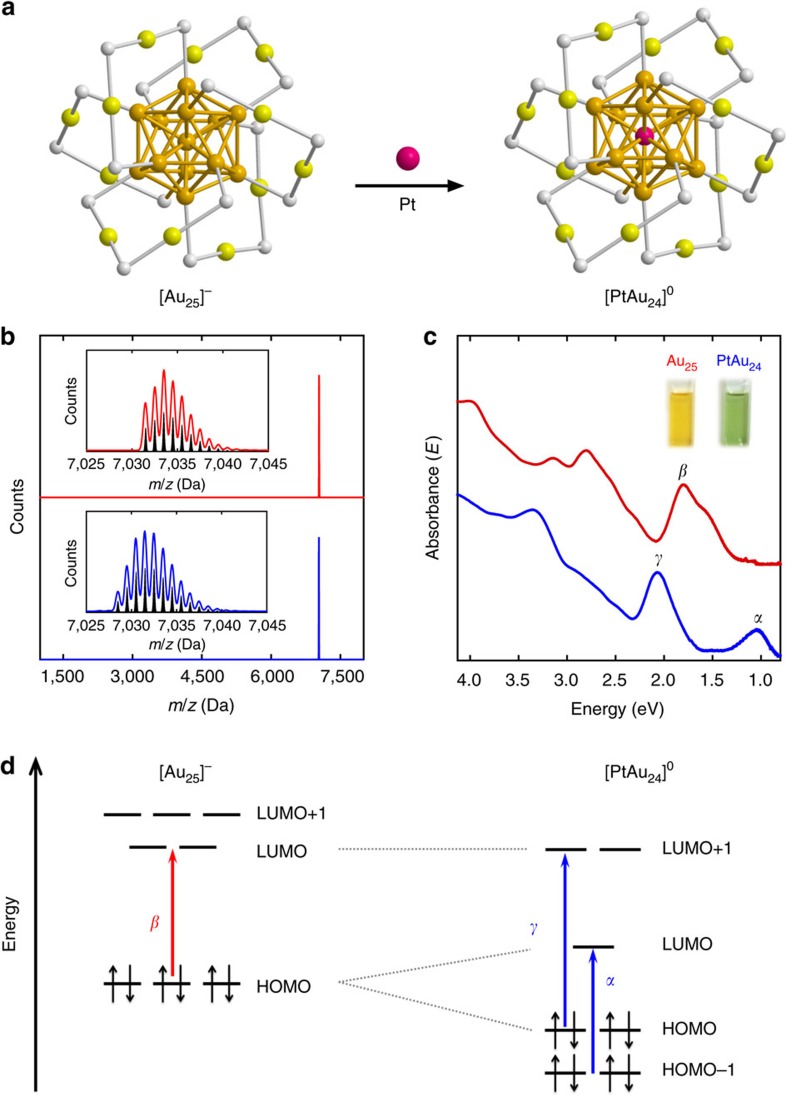
Characterizations of molecule-like metal clusters. (**a**) Structures of Au_25_ and PtAu_24_ clusters (golden, gold atoms of the core; olive, gold atoms of the shell; grey, sulfur (the rest of the ligand is omitted for clarity)). (**b**) Matrix-assisted laser desorption ionization (MALDI) mass spectra of Au_25_(SC_6_H_13_)_18_ (red) and PtAu_24_(SC_6_H_13_)_18_ (blue). The insets show the comparisons between the experimental data and the simulated isotope patterns (black sticks). (**c**) UV-vis-NIR absorption spectra of [Au_25_]^−^ (red) and [PtAu_24_]^0^ (blue) in tetrachloroethylene. The wavelength-scale absorption spectrum, Abs(*λ*), was converted to the energy-scale spectrum, absorbance (*E*), according to the relation Abs(*E*)∝[Abs(*λ*)]*λ*^2^. The insets show the photographs of [Au_25_]^−^ and [PtAu_24_]^0^ in CH_2_Cl_2_. (**d**) Electronic energy levels of [Au_25_]^−^ and [PtAu_24_]^0^. *α*, *β* and *γ* denote the optical transitions observed for [Au_25_]^−^ and [PtAu_24_]^0^ in **c**.

It has been found that Pt can selectively replace the central gold atom in the Au_25_ cluster, leading to a single Pt-doped bimetallic cluster (Fig. 1a)^30^,^33^,^34^. The Au_25_ and Pt-doped clusters were synthesized according to the procedures reported elsewhere^34^ (see Supplementary Notes 1–3 for details). Although they have been fully characterized in our previous report^34^, we will next briefly discuss their mass spectra, and optical and electrochemical properties, which can serve as an introduction to these molecule-like metal clusters. In Fig. 1b, the matrix-assisted laser desorption ionization mass spectra of the isolated gold (red) and Pt-doped gold clusters (blue) show only a single peak, indicating that the isolated clusters are extremely monodisperse. The peak at *m*/*z* ∼7,034 Da observed for the gold cluster (red) corresponds to the intact ion with a chemical composition of Au_25_(SC_6_H_13_)_18_. The peak at *m/z* ∼7,032 Da observed for the Pt-doped gold cluster is however almost superimposed with that of Au_25_(SC_6_H_13_)_18_ due to their small mass difference. The isotope pattern of the Pt-doped gold cluster (blue) shown in the inset of Fig. 1b can clearly be distinguished from that of Au_25_(SC_6_H_13_)_18_ (red) and matches well with the simulated isotope pattern (black) of PtAu_24_(SC_6_H_13_)_18_, manifesting that one Au atom in the Au_25_ cluster has been cleanly replaced by a Pt atom. Hereafter, these clusters will be abbreviated as Au_25_ and PtAu_24_, respectively. The average core size of both clusters is found to be around 1.1 nm (Supplementary Fig. 1).

The ultraviolet-vis-near-infrared (NIR) absorption spectra in Fig. 1c show distinctively different profiles of Au_25_ and PtAu_24_ clusters. The absorption profile is shown in the photon energy scale to show the broad NIR peak more clearly. The Au_25_ cluster exhibits the characteristic absorption features of Au_25_ clusters with peaks at 1.8, 2.8 and 3.1 eV. On Pt doping, the absorption profile changes drastically; that is, there is a new absorption band that appears at 2.1 eV with an additional NIR band centred at 1.1 eV in the low-energy region. The absorption profile of the Pt-doped cluster is consistent with that of neutral [PtAu_24_]^0^ where the Pt dopant is located at the centre of the core^33^. Evidently, replacing the central Au atom with Pt results in stable [PtAu_24_]^0^ having a superatomic 6-electron configuration^25^,^26^,^34^. The change in superatomic electronic configuration from 8-electron [Au_25_]^−^ to 6-electron [PtAu_24_]^0^ would lead to the splitting of the 1P orbital accompanying a Jahn–Teller-like distortion of the PtAu_12_ core^34^. The appearance of the new bands at 1.1 eV (*α*) and 2.1 eV (*γ*) and the disappearance of the band at 1.8 eV (*β*) for [PtAu_24_]^0^ in Fig. 1c,d are the results of the change in electronic structure of PtAu_24_.

### Electrochemistry and electrocatalytic activities

**Figure 2:**
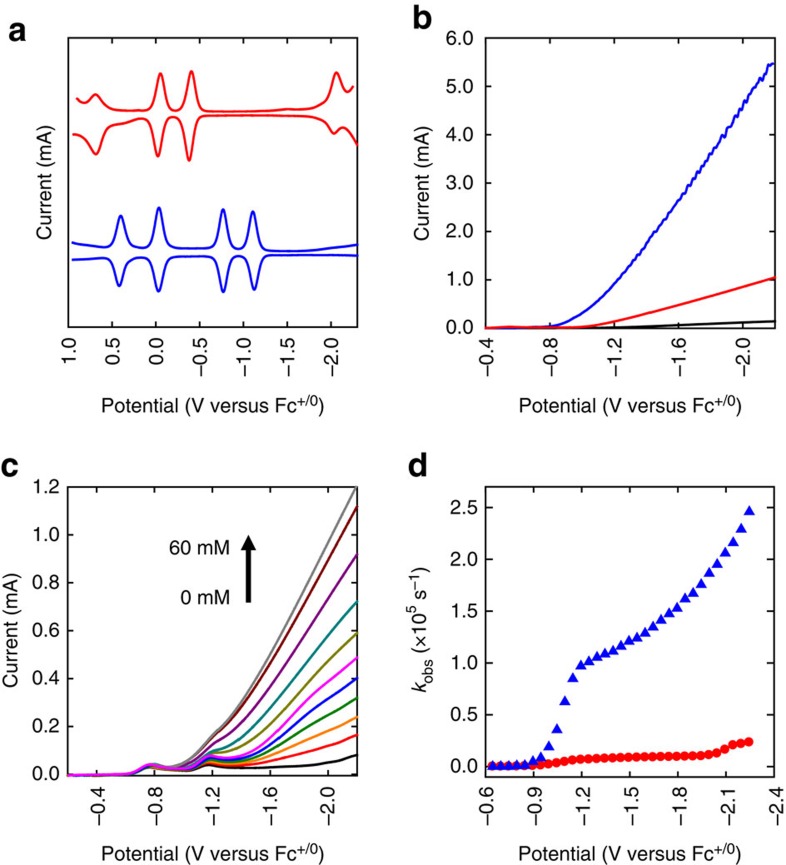
Voltammograms and electrocatalytic activities of metal clusters. (**a**) SWVs of Au_25_ (red) and PtAu_24_ (blue) in CH_2_Cl_2_ (0.1 M Bu_4_NPF_6_). (**b**) LSVs in THF (0.1 M Bu_4_NPF_6_) solution containing 1.0 M TFA in the absence (black) and presence of 1 mM Au_25_ (red) and 1 mM PtAu_24_ (blue) at 50 mVs^−1^. (**c**) LSVs of PtAu_24_ (1 mM) in THF (0.1 M Bu_4_NPF_6_) at 50 mVs^−1^ in the presence of 0, 4, 8, 12, 17, 21, 27, 34, 45, 55 and 60 mM of TFA. (**d**) *k*_obs_-potential plots for Au_25_ (red) and PtAu_24_ (blue) in the presence of 1.0 M TFA. For the precise comparison, measured potentials were corrected using ferrocene (Fc^+/0^) as an internal standard.

The optical measurements show that the electronic structure of Au_25_ cluster is drastically altered on doping of a Pt atom. To further unravel the electronic structure of the PtAu_24_ cluster, square-wave voltammetry (SWV) of Au_25_ and PtAu_24_ clusters were conducted. Voltammetry has been effectively used to study the electron transfer properties and the electronic structures of gold nanoclusters^19^,^34^. Understanding the redox behaviours near the HOMO-LUMO (highest occupied and lowest unoccupied molecular orbitals) levels is of particular importance in the design of efficient electrocatalysts. The SWVs in Fig. 2a exhibit well-resolved current peaks that lie at the formal potentials of the cluster charge-state couples. The open-circuit potential was found at −0.49 V for the Au_25_ cluster, indicating that the Au_25_ cluster is in anionic form, that is, [Au_25_]^−^. Therefore, the current peaks observed at −0.39 (O1), −0.04 (O2) and 0.69 (O3) correspond to the first, second and third oxidation peaks of the [Au_25_]^−^ cluster, while the peak at −2.06 V (R1) corresponds to the first reduction peak. The electrochemical gap determined from the difference between the first oxidation (O1) and reduction (R1) potentials is found to be 1.67 V. The HOMO-LUMO gap for [Au_25_]^−^ is determined to be 1.32 V by subtracting the charging energy term (O1–O2) of 0.35 V from the O1–R1 gap.

The redox potentials are drastically altered on doping of a Pt atom in the core as can be seen in Fig. 2a. Unlike the anionic Au_25_ cluster, PtAu_24_ is charge neutral with the open-circuit potential found at −0.49 V. Thus, the current peaks observed at −0.03 (O1) and 0.41 (O2) correspond to the first and second oxidation peaks of the PtAu_24_ cluster, while −0.76 (R1) and −1.10 V (R2) correspond to the first and second reduction peaks. The observed O1–R1 gap and the deduced HOMO-LUMO gaps from the SWV in Fig. 2a are found to be dramatically reduced to 0.73 V and 0.29 V, respectively. This result indicates that the electronic structure has indeed been greatly altered on Pt doping, and, more importantly, reduction potentials of [PtAu_24_]^0^ shifted positively by nearly 1 V compared to that of [Au_25_]^−^, potentially offering the possibility to lower overpotentials for reductive electrocatalysis.

Figure 2b shows linear sweep voltammograms (LSVs) in THF (0.1 M Bu_4_NPF_6_) solution containing 1.0 M trifluoroacetic acid (TFA) in the absence (black) and presence of Au_25_ (red) and PtAu_24_ (blue) clusters at a glassy carbon electrode (GCE). Compared to the blank current, the current for the proton reduction is significantly increased in the presence of Au_25_. The current further increases in the presence of PtAu_24_. The onset potential (*E*_onset_) of catalytic current is observed at −1.10 V for Au_25_, positively shifted by 150 mV compared to that of the blank GCE. In the presence of PtAu_24_, the onset was found at −0.89 V, positively shifted by 360 mV. The thermodynamic reduction potential of proton is estimated to be −0.82 V in THF (1.0 M TFA) and thus the onset potential of −0.89 observed for PtAu_24_ corresponds to an overpotential of 70 mV (refs 35, 36), which is comparable to that of natural hydrogenase (∼100 mV) enzymes^37^.

The catalytic activity of PtAu_24_ was further examined with increasing the concentration of TFA. As shown in Fig. 2c, the LSV clearly shows current peaks at −0.76 and −1.10 V corresponding to the first and second reduction of the PtAu_24_ cluster. Whereas there is no significant change observed for the [PtAu_24_]^0/−^ peak, the [PtAu_24_]^−/2−^ peak current drastically increases with TFA, suggesting that HER is greatly enhanced at the potential where [PtAu_24_]^2−^ is formed. That the HER current increases at the potential of the [PtAu_24_]^−/2−^ couple strongly suggests that PtAu_24_ acts as an electron transfer mediator for HER that shuttles electrons from GCE to proton in the solution^38^,^39^. Interestingly, the onset potential of the second reduction of PtAu_24_ matches well with the *E*_onset_ (−0.89 V) observed in Fig. 2b, suggesting that the HER catalytic activity is indeed dependent on the charge state of PtAu_24_. When the catalytic activity of Au_25_ was examined with increasing concentration of TFA, the current increase at a more negative potential (−1.1 V) and the reduction current associated with HER was found to be much smaller (Supplementary Fig. 2). This result unambiguously shows that Pt doping significantly alters the redox potentials of the host cluster and thus drastically enhance its catalytic activity. In addition, the charge-state-dependent catalytic activity of PtAu_24_ clusters indicates that they are like molecular catalysts that carry discrete charge for reaction, which sets these clusters apart from other metal nanoparticle systems.

At sufficiently high acid concentration relative to the catalyst, the following equation can be used to calculate pseudo-first-order rate constant, *k*_obs_, for H_2_ evolution that is catalysed by freely diffusing catalysts:^6^,^13^,^40^


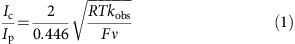


where *I*_c_ is the catalytic current, *I*_p_ is the peak current in the absence of acid (here taken from the wave of [PtAu_24_]^0/−^ or [Au_25_]^0/−^), 2 is the number of electrons involved in the catalytic reaction, *R* is the ideal gas constant, *T* is the temperature in Kelvin, *F* is Faraday's constant and *v* is the scan rate. As can be seen in Supplementary Fig. 3a, *I*_c_ increases with increasing acid concentration and levels off above 1.0 M TFA. At the acid concentration of 1.0 M, *k*_obs_ was then obtained by plotting *I*_c_/*I*_p_ as a function of *v*^−1/2^ as shown in Supplementary Fig. 3b. The *k*_obs_ value calculated from the slope of the fit line (Supplementary Fig. 3b) is 121,000 s^−1^ at −1.5 V (*η*=650 mV). Under the same experimental condition, the *k*_obs_ value calculated in the presence of Au_25_ is only 8,000 s^−1^. The rate constant observed for PtAu_24_ is also significantly higher than those reported to date for highly active molecular electrocatalysts; compared to cobalt complexes (700 s^−1^ at *η*=890 mV)^41^ and copper complexes (11,000 s^−1^ at *η*=720 mV)^13^ in comparable conditions, it is ca. 170- and 11-fold higher, respectively. To the best of our knowledge, the highest *k*_obs_ value (106,000 s^−1^) thus far reported was observed at *η*=650 mV for a nickel complex that catalyses H_2_ formation with pendant amines that act as proton relays^6^. The *k*_obs_ value obtained for PtAu_24_ is also higher than that.

To understand the origin of the extraordinary catalytic activity observed for PtAu_24_, we compared *k*_obs_ as a function of potential. As can be seen in Fig. 2d, the *k*_obs_ value found at −0.89 V is 1,300 s^−1^ and sharply increases to 62,000 s^−1^ at −1.1 V, followed by a gradual increase to 186,000 s^−1^ at −2.0 V. Interestingly, the potential where the drastic increase occurs matches well with the second reduction wave [PtAu_24_]^−/2−^. By contrast, the *k*_obs_ value calculated for Au_25_ is found to be relatively small (<16,000 s^−1^) until −2.1 V, where [Au_25_]^−^ is reduced to [Au_25_]^2−^.

### Mechanisms of HER

**Figure 3:**
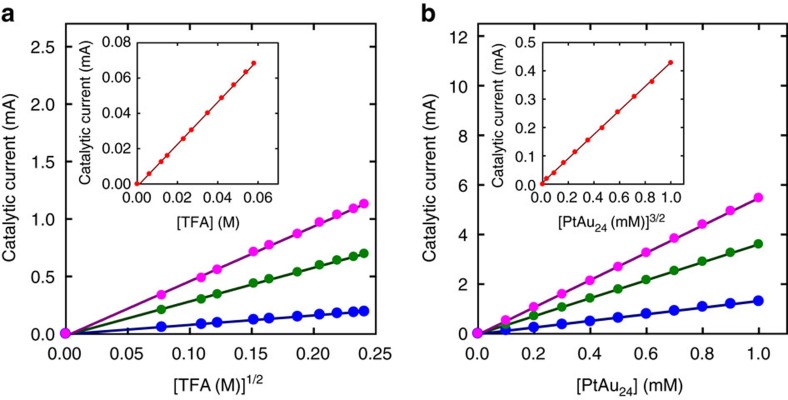
Charge-state-dependent electrochemical HER mechanisms catalysed by PtAu_24_. Dependence of the catalytic current, *I*_c_, (**a**) on the concentration of TFA in the presence of PtAu_24_ (1 mM) and (**b**) on the concentration of PtAu_24_ in TFA (1.0 M) solution at −1.3 (blue), −1.8 (green) and −2.2 V (purple). Insets show plots for dependence of the *I*_c_ on the concentration of (**a**) TFA and (**b**) PtAu_24_ at −1.0 V. The data are fitted by first-order linear functions (solid lines).

The mechanism study shown in Fig. 3 clearly exhibits the charge-state-dependent catalytic activity of PtAu_24_. That is, the catalytic currents observed at potentials negative to the [PtAu_24_]^−/2−^ wave (that is, −1.3, −1.8 and −2.2 V) all exhibit linear correlation with [PtAu_24_] and [TFA]^1/2^, respectively, which is consistent with the heterolytic HER mechanism (equation (3))^42^,^43^. On the other hand, at −1.0 V where PtAu_24_ is predominantly present in the form of [PtAu_24_]^−^, the currents exhibit linear correlation with [TFA] and [PtAu_24_]^3/2^, respectively, as shown in Fig. 3 insets, indicating the homolytic HER mechanism (equation (4))^42^.


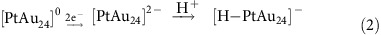


Heterolytic pathway:


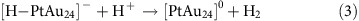


Homolytic pathway:


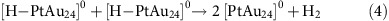


This result is very reasonable considering the charge state of PtAu_24_. [PtAu_24_]^2−^ reacts with proton to form [H-PtAu_24_]^−^ intermediate that is negatively charged and thus preferably react with proton to evolve H_2_. On the other hand, at −1.0 V [PtAu_24_]^−^ reacts with proton to form [H-PtAu_24_]^0^ intermediate that favours homolytic HER pathway. Similar charge-state dependence is observed for Au_25_. As shown in Supplementary Fig. 4, the catalytic currents exhibit linear correlation with [TFA] and [Au_25_]^3/2^, respectively, indicating homolytic pathway, at −1.0, −1.3 and −1.8 V, where Au_25_ clusters are present in the form of [Au_25_]^−^. When the cluster is reduced to [Au_25_]^2−^ at −2.2 V, the currents exhibit linear correlation with [TFA]^1/2^ and [Au_25_], respectively, as shown in the insets of Supplementary Fig. 4, indicating the heterolytic HER pathway. The charge-state-dependent catalytic activity has been observed for electrocatalysis using Co complexes where [Co(II)H]^−^ favours heterolytic HER pathway, while homolytic pathway is favoured by [Co(III)H]^0^ intermediate^44^. The charge-state-dependent catalytic activity of PtAu_24_ observed in this work clearly display the characteristic of molecular catalysts.

The charge state is not the only factor affecting the HER activity of the clusters. Comparing [PtAu_24_]^2−^ with [Au_25_]^2−^, the *k*_obs_ value calculated for [PtAu_24_]^2−^ is 229,000 s^−1^, more than 10-fold higher than that of [Au_25_]^2−^ (22,000 s^−1^) at −2.2 V at which both clusters are present as a dianionic form. The vastly different catalytic activity can be understood by considering the reduction potential match between the catalyst and the substrate (proton). That is, when the reduction potential of catalyst matches closely with the thermodynamic potential of proton, the catalyst can act as an effective electron transfer mediator and enhance its activity dramatically^41^,^45^,^46^. In this work, the reduction potential of catalyst can be precisely tuned to match with the potential of HER by Pt doping, enabling judiciously optimized electrocatalysis.

**Figure 4:**
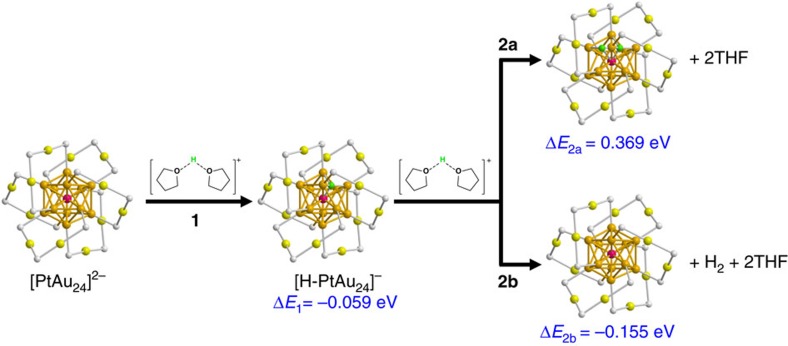
Calculated reaction energies for H_2_ evolution on PtAu_24_. In step 1, a solvated proton is transferred from THF molecules to [PtAu_24_]^2−^ to form [H-PtAu_24_]^−^; in step 2a, a second solvated proton is transferred from THF molecules to [H-PtAu_24_]^−^ to form [2H-PtAu_24_]; in step 2b, a second solvated proton reacts with H in [H-PtAu_24_]^−^ to form H_2_. The calculations are at the DFT-TPSS level; besides the two explicit solvent molecules, an implicit solvent model is also included for the whole system (see ‘Methods' section). Colour code for the cluster structure: golden, Au atoms of the core; olive, Au atoms of the shell; purple, Pt atom; green, adsorbed H from the liquid medium; grey, S (the rest of ligand is omitted for clarity).

To gain further insight into the origin of the extraordinary catalytic activity of PtAu_24_, we have compared the hydrogen adsorption and the HER energetics calculated using density functional theory (DFT) for each cluster. We modelled the HER process by using protons solvated by two THF molecules, which are then adsorbed on the cluster surface; the whole system is further solvated by an implicit solvation model. Figure 4 shows that the energy change of the Volmer step (step 1) calculated for [PtAu_24_]^2−^ is rather thermodynamically neutral (−0.059 eV), whereas that for [Au_25_]^−^ is 0.539 eV. This result clearly suggests the initial hydrogen binding is energetically favourable on [PtAu_24_]^2−^, but is endothermic on [Au_25_]^−^, explaining the high HER activity observed for [PtAu_24_]^2−^. However, the second H adsorption on [PtAu_24_]^2−^ and [Au_25_]^−^ is found to be both endothermic; 0.369 and 1.21 eV for [H-PtAu_24_]^−^ (step 2a in Fig. 4) and [H-Au_25_]^0^, respectively. Thus, the DFT calculations predict that hydrogen evolution occurs on PtAu_24_ via the coupling of the adsorbed hydrogen with another proton from the solution (that is, heterolytic or Heyrovsky pathway) with an energy change of −0.155 eV (step 2b in Fig. 4). By contrast, the most thermodynamically favourable H_2_ generation occurs on Au_25_ via the homolytic pathway (Supplementary Fig. 5). These calculation results are indeed in consistent with the experimentally found HER mechanisms for [PtAu_24_]^2−^ and [Au_25_]^−^. The idea of thermoneutral catalysts for HER regarding hydrogen binding has been demonstrated experimentally for pure metals, alloys and layered solid catalysts^16^,^17^,^18^,^47^. Recently, it has been pointed out that this concept can be applied to molecular complexes, although it is significantly difficult to find the thermoneutral complexes because of their inherently complex chemical environment^17^,^35^,^48^. In this work, we have demonstrated that the redox potentials and binding affinity can be fine-tuned by simple doping of a Pt atom into gold catalysts, opening a new avenue to the fine-tuning of the catalytic properties.

Figure 4 also shows that the hydrogen preferably binds on the hollow site of the surface of the PtAu_12_ core. It is interesting to note that the bond distance between the central Pt and the adsorbed H is found to be 1.788 Å, significantly shorter than the distance between the surface Au and the adsorbed H (2.031 Å), indicating that the adsorbed H atom forms H-Pt chemical bond with the central Pt. In the DFT calculations, we initially placed the H atom on the intact cluster surface away from the Au atoms; during geometry optimization, we found that the H atom spontaneously moves into the subsurface to interact directly with the central Pt atom while breaking some surface Au-Au bonds. This result suggests that the H-Pt bond formation is a downhill process with no transition state. The H-Pt bond is also manifested in the local electronic density of states (Supplementary Fig. 6) that shows the hybridization between Pt 5*d* states and the H 1*s* state. The stronger H–Pt interaction than the H–Au interaction is a key factor contributing to the favourable HER energetics (Fig. 4) on [PtAu_24_]^2−^ than on [Au_25_]^−^.

### Electrocatalytic H_2_ production

**Figure 5:**
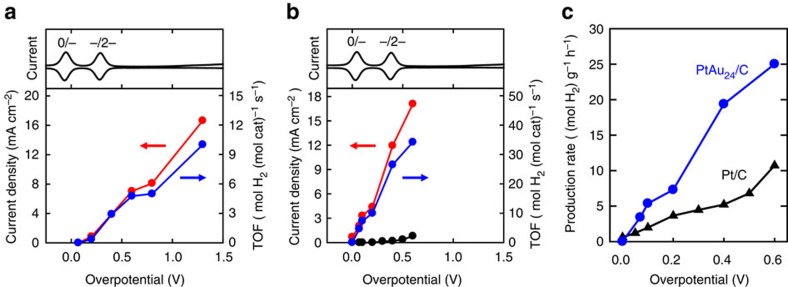
CPE data for PtAu_24_ in homogeneous and heterogeneous conditions. (**a**) Average current densities (red circles) and TOFs (blue circles) obtained at various overpotentials after 15 min homogeneous CPE in THF (0.5 M Bu_4_NPF_6_) containing 1.0 TFA with a glassy carbon plate (1 cm^2^) in the presence of PtAu_24_ (2.5 μM). (**b**) Average current densities (red circles) and TOFs (blue circles) obtained at various overpotentials after 60 min heterogeneous CPE in 1.0 M Brinton–Robinson buffer solution (pH 3) on a PtAu_24_/C/GDL electrode (1 cm^2^). Average current densities obtained on a blank C/GDL (black circles) are shown for comparison. SWVs of PtAu_24_ shown in the upper part of the graphs show the charge state of PtAu_24_ at each overpotential. (**c**) H_2_ production rates per mass of metals in the catalyst ((mol H_2_)  g^−1^ h^−1^) at various overpotentials on a PtAu_24_/C/GDL (blue circles) and a Pt/C/GDL (black triangles) electrodes (1 cm^2^).

To verify the catalytic production of H_2_, we carried out controlled potential electrolysis (CPE) with PtAu_24_ in THF containing 1.0 M TFA in an H-type cell. Figure 5a shows plots of the average current density and the amount of H_2_ detected by gas chromatography analysis at each overpotential. The clusters display symptoms of decomposition after prolonged CPE experiments and thus the electrolysis was conducted for 15 min. As can be seen in the figure, production of H_2_ is first detected at an overpotential of 0.2 V and increases with increasing overpotential. Comparison of the amount of H_2_ produced with the charge consumed indicates that the current efficiency for H_2_ production is > 97% when the overpotential is 0.4 V or higher. After subtracting the H_2_ production from the blank solution, the TOF at *η*=0.6 V is found to be 4.8 mol H_2_ (mol cat)^−1^ s^−1^. To the best of our knowledge, this value is considerably higher than any other molecular catalysts reported for HER in similar conditions^6^,^7^,^8^,^9^,^12^,^13^. The highest TOF value reported thus far is 4.1 mol H_2_ (mol cat)^−1^ s^−1^ obtained for a copper complex at a higher overpotential of 0.75 V (ref. 13). The nickel complex exhibiting a *k*_obs_ value of 106,000 s^−1^ shows a TOF of 0.24 mol H_2_ (mol cat)^−1^ s^−1^ at *η*=0.92 V (ref. 6).

While PtAu_24_ exhibits high catalytic activity for H_2_ production with moderate overpotential requirements in a non-aqueous solvent, it would be more practical if it can be immobilized on electrode materials that can fully operate in aqueous media. There are a number of immobilization strategies available for gold nanoparticles via their surface functionalization^39^,^49^,^50^. In this work, PtAu_24_ cluster solution mixed with a carbon black (C) and Nafion was dropcast on a gas diffusion layer (GDL) electrode (PtAu_24_/C/GDL). We found that the clusters immobilized on the carbon black with the Nafion binder exhibit higher stability during electrolysis. Figure 5b shows plots of the average current density and the amount of H_2_ detected after 60 min CPE at each overpotential in 1.0 M Brinton–Robinson buffer solution (pH 3). As can be seen in the figure, H_2_ generation was achieved near the thermodynamic potential (*η*=70 mV), which also matches very well with the *E*_onset_ found in Fig. 2b. The charge passed and H_2_ generation increased rapidly with increasing overpotential and the current efficiency for HER went up to nearly 100% when the overpotential is 100 mV or higher. At *η*=400 mV, the average current density increases above 12 mA cm^−2^. Having immobilized such well-defined clusters on the electrode, we were able to calculate the TOF from the CPE experiments. As can be seen in Fig. 5b, the TOF values obtained from the heterogeneous catalysis are remarkably high; 34 mol H_2_ (mol cat)^−1^ s^−1^ at *η*=0.6 V in aqueous media after subtracting the contribution from the blank C/GDL electrode. This value is again much higher than those obtained in comparable conditions. For comparison, a TOF of 2.2 mol H_2_ (mol cat)^−1^ s^−1^ was obtained at *η*=0.59 V in acetate buffer (pH 4.5) for a cobalt catalyst immobilized on carbon nanotubes^14^.

Although it is not straightforward to directly compare the TOF values obtained from the homogeneous (Fig. 5a) and heterogeneous (Fig. 5b) catalysis, the enhanced TOF obtained in the latter in aqueous media is highly encouraging and may reflect the fact that all clusters on the electrode are available for catalysis. The high catalytic activity observed for the PtAu_24_/C/GDL electrode has prompted us to compare its activity with the benchmarking Pt/C (commercial 20 wt% Pt on Vulcan carbon black) dropcast on a GDL electrode (1 cm^2^). Since TOF could not be determined for the Pt/C, we have compared the H_2_ production rate per mass of metals (that is, Au+Pt) in the catalyst composites (mol H_2_ g^−1^ h^−1^). As can be seen in Fig. 5c, both PtAu_24_/C/GDL and Pt/C/GDL electrodes start to produce H_2_ near the thermodynamic potential, but the H_2_ production rate determined for the PtAu_24_/C/GDL is much higher than that for the Pt/C/GDL. For instance, the H_2_ production rate determined for the PtAu_24_/C/GDL is 25 mol H_2_ g^−1^ h^−1^, which is more than two times higher than that for the Pt/C/GDL catalyst (11 mol H_2_ g^−1^ h^−1^) at the same overpotential of 0.6 V.

## Discussion

Here we show that the electronic structure and the catalytic activity of a cluster catalyst can be fine controlled by doping a Pt atom into a stable gold cluster. The bimetallic cluster is molecule-like and exhibits excellent catalytic activity for H_2_ production; significantly higher than any other molecular catalysts reported thus far, to the best of our knowledge, and even higher than the benchmarking platinum catalyst. Mechanistic investigations have revealed that hydrogen binding step on the bimetallic cluster is thermodynamically neutral and the central Pt atom forms Pt-H chemical bond, pointing to a key role of the dopant. The molecule-like bimetallic cluster operates efficiently in both homogeneous and heterogeneous conditions, representing a special type of electrocatalyst. The molecule-like bimetallic cluster may thus provide a platform for the discovery of finely tuned catalysts, which could have broad implications for catalysis beyond H_2_ production.

## Methods

### Electrochemical methods

SWV and LSV were conducted with an electrochemical workstation (model 660B; CH Instruments) in CH_2_Cl_2_ (SWV) or THF (LSV) containing 0.1 M Bu_4_NPF_6_ as a supporting electrolyte that was degassed and blanketed with a high-purity Ar gas. A GCE (3 mm diameter) was used as a working electrode, a Pt wire as the counter electrode and Ag/AgNO_3_ (0.1 M AgNO_3_ in CH_3_CN) as the reference electrode for the LSV experiments. SWV was carried out with a Pt disk (0.4 mm diameter) working electrode at 100 mV s^−1^ with a pulse height and a width of 20 mV and 20 ms, respectively. Ferrocene (Fc^+/0^) was added as an internal reference for Ag/AgNO_3_.

### Controlled potential electrolysis

CPE experiments in homogeneous condition were conducted in a 5 ml cell containing 2.5 μM cluster and 1.0 M TFA dissolved in 2.0 ml THF (0.5 M Bu_4_NPF_6_) that was degassed and blanketed with Ar gas. Electrolysis was conducted for 15 min under vigorous stirring with an electrochemical workstation (model 660B; CH Instruments) equipped with three electrodes consisting of glassy carbon plate working electrode (area=1 cm^2^), a GDL (model N1S1007; CeTech Co., Taiwan) counter electrode (1 cm^2^) and a Ag/AgNO_3_ reference electrode. CPE experiments in heterogeneous condition were carried out for 60 min under vigorous stirring with a ZIVE MP1 potentiostat (WonATech, Korea) in an H-type cell that was equipped with a composite working electrode and a Ag/AgCl (3 M NaCl) reference electrode in one compartment and a platinum plate (1.68 cm^2^) counter electrode in the other compartment. The working electrode was separated from the counter electrode by a proton exchange membrane (Nafion 117; Sigma-Aldrich). Both compartments were filled with 60 ml of 1.0 M Brinton–Robinson buffer (pH 3.0) that was degassed and blanketed with Ar gas. The composite working electrode was fabricated by spreading a catalyst ink prepared by mixing 16 μg of the cluster catalyst, 200 μg of carbon black (Vulcan XC-72) and 1.5 μl of Nafion solution (5 wt%; Sigma-Aldrich) in 50 μl of THF on a GDL (N1S1007; CeTech Co.) electrode (1 cm^2^). For comparison, CPE experiments were conducted under the same condition using a commercial Pt/C (20 wt% platinum on Vulcan carbon black; Sigma-Aldrich) catalyst that was mixed with Nafion and dropcast on a GDL electrode (1 cm^2^). The average size of Pt particle was 3.5±0.5 nm. The amount of H_2_ evolved was quantified from an analysis of the headspace using an Agilent 7890B gas chromatography equipped with a thermal conductivity detector.

### Computational methods

DFT calculations of the HER on Au_25_(SCH_3_)_18_ and PtAu_24_(SCH_3_)_18_ clusters were performed with the quantum chemistry program Turbomole V6.5. The def2-SV(P) basis sets were used for C, S and H, while effective core potentials which include scalar relativistic corrections and 19 (18) valence electrons were used for Au (Pt). Geometry optimization was done with the TPSS (Tao, Perdew, Staroverov and Scuseria) functional. In our calculations, solvent effects were treated implicitly by making use of the conductor-like screening model (COSMO) as implemented in Turbomole.

### Data availability

The authors declare that all the other data supporting the findings of this study are available within the article and its Supplementary Information Files or from the corresponding author on reasonable request.

## Additional information

**How to cite this article:** Kwak, K. *et al*. A molecule-like PtAu_24_(SC_6_H_13_)_18_ nanocluster as an electrocatalyst for hydrogen production. *Nat. Commun.* **8,** 14723 doi: 10.1038/ncomms14723 (2017).

**Publisher's note**: Springer Nature remains neutral with regard to jurisdictional claims in published maps and institutional affiliations.

## Supplementary Material

Supplementary Information Supplementary Figures and Supplementary Notes
