# Supplementary material for: A molecule-like PtAu24(SC6H13)18 nanocluster as an electrocatalyst for hydrogen production
Source: Nat Commun. 2017 Mar 10;8:14723. doi: 10.1038/ncomms14723 (PMC5353570; doi:10.1038/ncomms14723)
Supplement: Supplementary Information — Supplementary Figures and Supplementary Notes [file ncomms14723-s1.pdf]

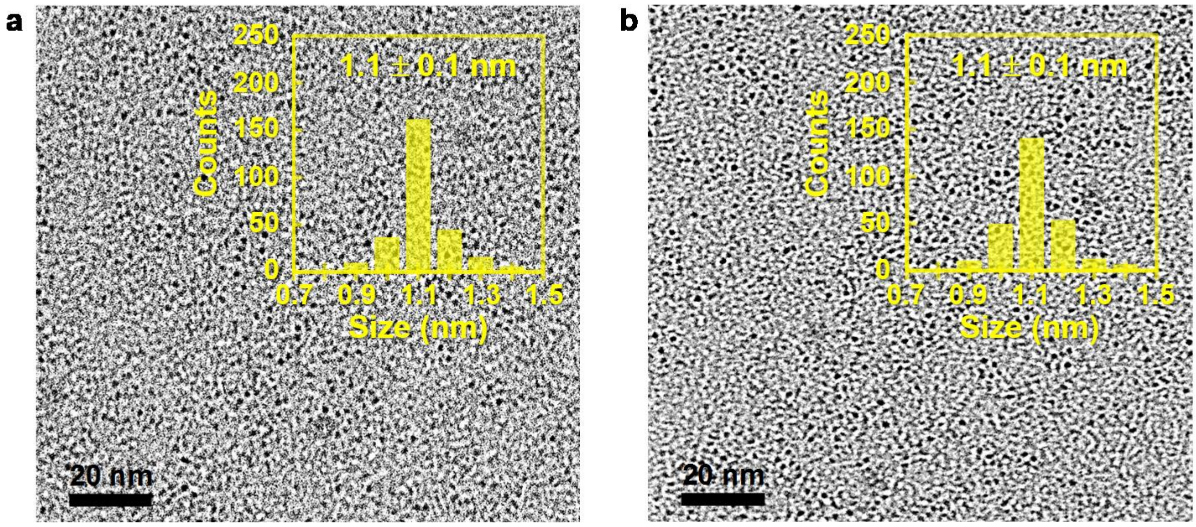


**Supplementary Figure 1 | Transmission electron microscopy (TEM) images of metal clusters.** TEM images of **a,** $Au_{25}$ and **b,** $PtAu_{24}$ clusters. Scale bar for the TEM images is 20 nm and the insets show the histograms of the core size distribution.

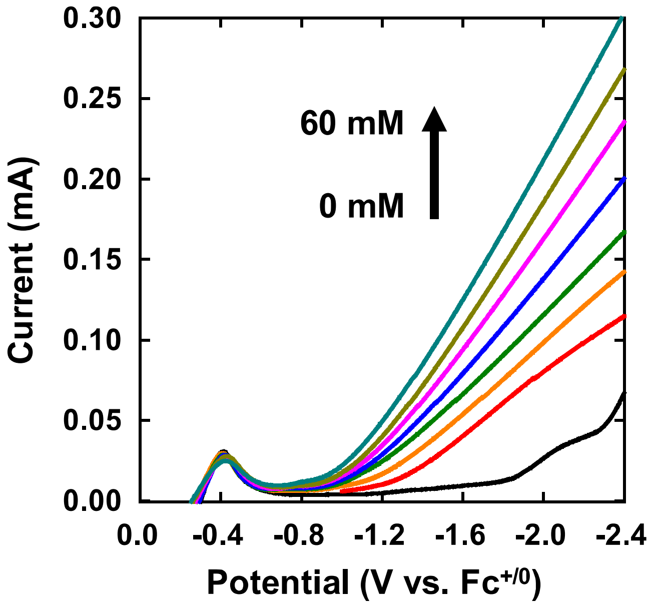


**Supplementary Figure 2 | TFA dependent HER catalyzed by $Au_{25}$.** LSVs of $Au_{25}$ (1 mM) in THF containing 0.1 M $Bu_4NPF_6$ at 50 $mVs^{-1}$ in the presence of 0, 4, 8, 12, 21, 34, 45, and 60 mM of TFA.

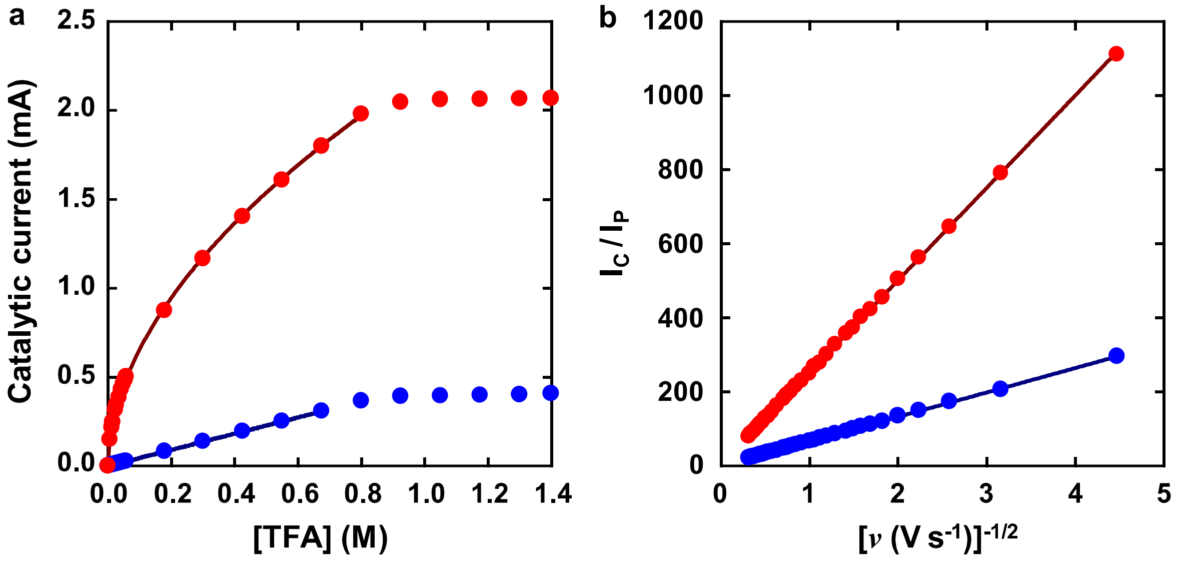


**Supplementary Figure 3 | Determination of $k_{obs}$ values. a,** Dependence of the catalytic current on the concentration of TFA in the presence of 1 mM $PtAu_{24}$ (red) and 1 mM $Au_{25}$ (blue) at -1.5 V. **b,** Plots of $I_c/I_p$ versus $\nu^{-1/2}$ ($\nu$ = 0.05 - 10 $Vs^{-1}$) for a THF (0.1 M $Bu_4NPF_6$) solution containing 1.0 M TFA in the presence of 1 mM $PtAu_{24}$ (red) and 1 mM $Au_{25}$ (blue) at -1.5 V.

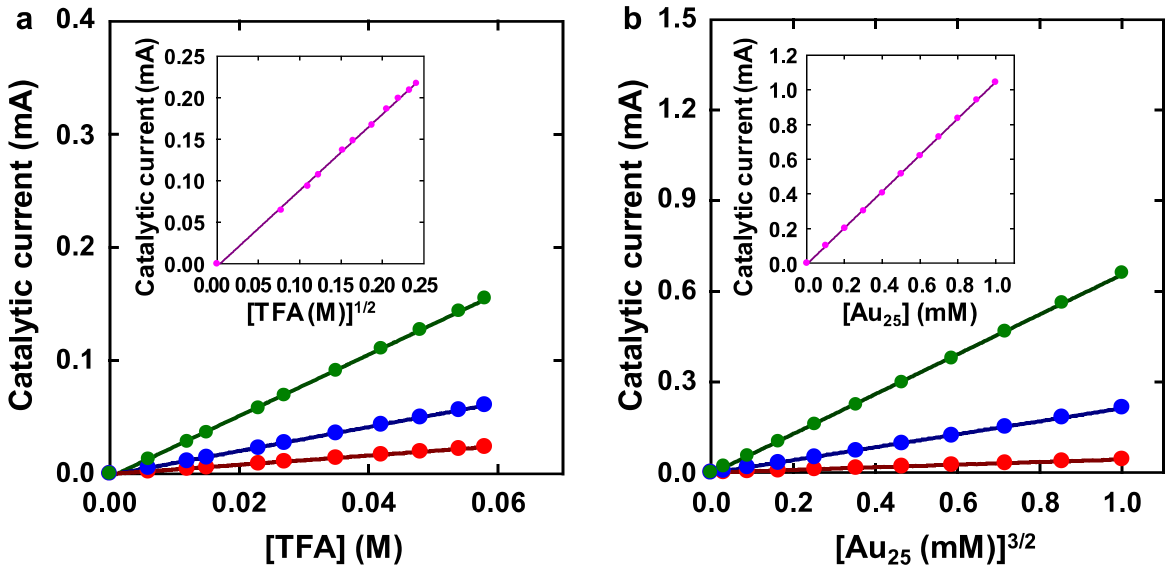


**Supplementary Figure 4 | Charge-state dependent electrochemical HER mechanisms catalyzed by $Au_{25}$.** Dependence of the catalytic current, $I_c$, **a,** on the concentrations of TFA in the presence of $Au_{25}$ (1 mM) and **b,** on the concentration of $Au_{25}$ in TFA (1.0 M) solution at -1.0 (red), -1.3 (blue), and -1.8 (green) V. Insets show plots for dependence of the $I_c$ on the concentrations of **a,** TFA and **b,** $Au_{25}$ at -2.2 V. The data are fitted by first-order linear functions (solid lines).

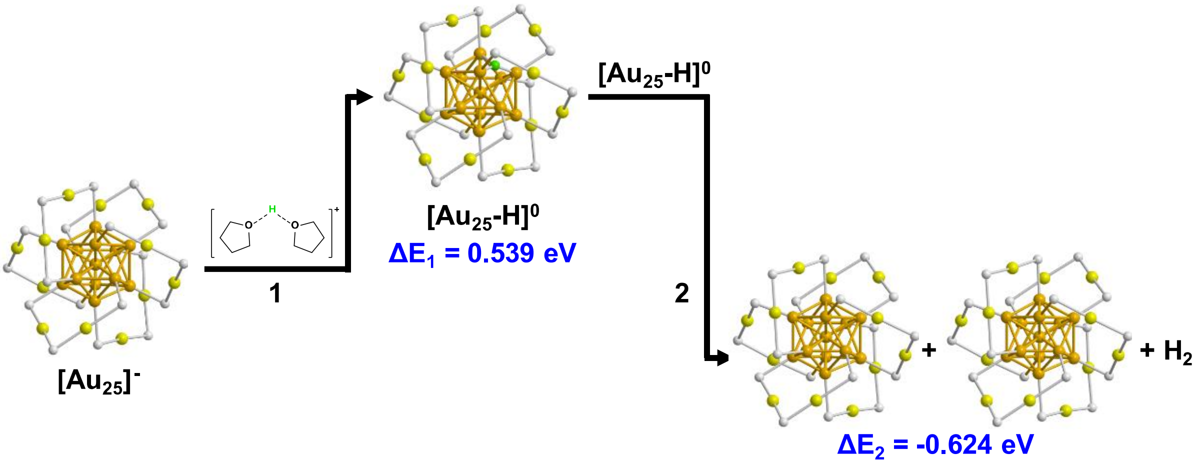


**Supplementary Figure 5 | Reaction pathway for the production of $H_2$ from the reaction of [$Au_{25}$]$^-$ with TFA.** In step 1, a solvated proton is transferred from THF molecules to [$Au_{25}$]$^-$ to form [H-$Au_{25}$]$^0$; in step 2, an adsorbed H in one [H-$Au_{25}$]$^0$ reacts with a second adsorbed H in a second [H-$Au_{25}$]$^0$ to form $H_2$. The calculations are at the DFT-TPSS level; besides the two explicit solvent molecules, an implicit solvent model is also included for the whole system.

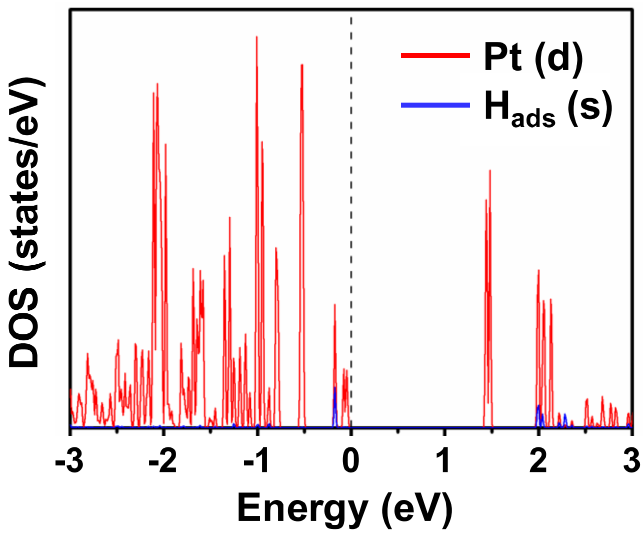


**Supplementary Figure 6 | The orbital-projected density of states on Pt in $PtAu_{24}$ and the adsorbed H atom.** The Fermi level is set as zero.

**Supplementary Note 1. Synthesis of $Au_{25}(SC_6H_{13})_{18}$ and $PtAu_{24}(SC_6H_{13})_{18}$.** The $Au_{25}$ and Pt-doped clusters were synthesized according to the procedures reported elsewhere (ref. 34). The syntheses were very reproducible and the purity of the synthesized clusters was confirmed by mass spectrometry and absorption spectrometry.

**Supplementary Note 2. Chemicals.** Potassium chloride (KCl, >99%), tetrabutylammonim hexafluorophosphate ($Bu_4NPF_6$, >99%), trifluoroacetic acid (TFA, >99%), Nafion® (5 wt% solution), and carbon black (Vulcan XC-72) were purchased from Sigma-Aldrich. Britton-Robinson aqueous universal buffer solution was prepared from phosphoric acid ($H_3PO_4$, 85%), boric acid ($H_3BO_3$, >98.5%), acetic acid ($CH_3COOH$, 99.5%), and sodium hydroxide (NaOH, 98%). Extrapure grade dichloromethane ($CH_2Cl_2$) and tetrahydrofuran (THF) were used. Water was purified using a Millipore Milli-Q system (18.2 MΩ·cm). All the chemicals were used as received without further purification.

**Supplementary Note 3. Characterization of metal clusters.** Matrix-assisted laser desorption ionization (MALDI) mass spectra were acquired using an AB Sciex MALDI-TOF mass spectrometer (4800 plus) equipped with a standard UV nitrogen laser (337 nm). The accelerating voltage was held at 15 kV and the spectrum was collected in a linear-positive ion mode. Sample solution in $CH_2Cl_2$ (0.7 mM) was mixed with trans-2-[3-(4-tert-butylphenyl)-2-methyl-2-propenylidene] malononitrile (DCTB) as a matrix (saturated in $CH_2Cl_2$) and then applied to the sample plate and air-dried. Absorption spectra were obtained from a Shimadzu UV-vis-NIR spectrophotometer (UV-3600) using freshly prepared cluster solutions in tetrachloroethylene. Transmission electron microscopy (TEM) images were recorded with a JEOL transmission electron microscope (JEOL 2100F). Samples for TEM were prepared by drop-casting $CH_2Cl_2$ solution of clusters (0.5 mg/mL) on a 400 mesh Formvar/carbon-coated copper grid (01814-F, Ted Pella) and drying for 1 h at room temperature before imaging.
